# Hunting, Swimming, and Worshiping: Human Cultural Practices Illuminate the Blood Meal Sources of Cave Dwelling Chagas Vectors (*Triatoma dimidiata*) in Guatemala and Belize

**DOI:** 10.1371/journal.pntd.0003047

**Published:** 2014-09-11

**Authors:** Lori Stevens, M. Carlota Monroy, Antonieta Guadalupe Rodas, Patricia L. Dorn

**Affiliations:** 1 Department of Biology, College of Arts and Sciences, University of Vermont, Burlington, Vermont, United States of America; 2 Escuela de Biologia, Universidad de San Carlos de Guatemala, Ciudad de Guatemala, Guatemala; 3 Department of Biological Sciences, Loyola University New Orleans, New Orleans, Louisiana, United States of America; Universidad Autónoma de Yucatán, Mexico

## Abstract

**Background:**

*Triatoma dimidiata*, currently the major Central American vector of *Trypanosoma cruzi*, the parasite that causes Chagas disease, inhabits caves throughout the region. This research investigates the possibility that cave dwelling *T. dimidiata* might transmit the parasite to humans and links the blood meal sources of cave vectors to cultural practices that differ among locations.

**Methodology/Principal Findings:**

We determined the blood meal sources of twenty-four *T. dimidiata* collected from two locations in Guatemala and one in Belize where human interactions with the caves differ. Blood meal sources were determined by cloning and sequencing PCR products amplified from DNA extracted from the vector abdomen using primers specific for the vertebrate 12S mitochondrial gene. The blood meal sources were inferred by ≥99% identity with published sequences. We found 70% of cave-collected *T. dimidiata* positive for human DNA. The vectors had fed on 10 additional vertebrates with a variety of relationships to humans, including companion animal (dog), food animals (pig, sheep/goat), wild animals (duck, two bat, two opossum species) and commensal animals (mouse, rat). Vectors from all locations fed on humans and commensal animals. The blood meal sources differ among locations, as well as the likelihood of feeding on dog and food animals. Vectors from one location were tested for *T. cruzi* infection, and 30% (3/10) tested positive, including two positive for human blood meals.

**Conclusions/Significance:**

Cave dwelling Chagas disease vectors feed on humans and commensal animals as well as dog, food animals and wild animals. Blood meal sources were related to human uses of the caves. We caution that just as *T. dimidiata* in caves may pose an epidemiological risk, there may be other situations where risk is thought to be minimal, but is not.

## Author Summary

Caves have enduring appeal to humans, and their lure in Central America includes tourism, religious ceremonies and shelter. The major Chagas disease vector in this region, *Triatoma dimidiata*, inhabits caves throughout its range. We challenge the assumption that cave-dwelling vectors are not important for human transmission by determining blood meal sources of vectors collected in caves from three locations that differ in the activities of humans at the caves, and link the results to cultural practices that differ among locations. Seventy percent of cave-collected vectors were positive for human DNA, and fed on 10 additional vertebrates with relationships to humans varying from companion animal (dog), food animals (pig, sheep/goat), wild animals (duck, bat, opossum) to commensal animals (mouse, rat). Feeding sources relate to human activities that vary among locations, for example, human and food animals were the main sources in Cahabón caves, located within deforested areas that are now agricultural fields and houses. We tested vectors from one location for infection with the Chagas disease parasite and found 30% (3 of 10) infected, including two of the three vectors from this location that had evidence of human blood. Humans should be aware of potential consequences of visiting and sleeping in caves.

## Introduction

Humans have been attracted to caves for much of our history for purposes as varied as religious ceremonies to simply shelter. In Guatemala people use caves for several cultural practices including religious ceremonies, tourism, and shelter [1]. In the department of Petén in northern Guatemala, caves are often used by hunters for sleeping and shelter from rain, especially in the rainy season. Also in northern Guatemala, caves in the department of Alta Verapaz are sacred places and sometimes the site of Mayan religious ceremonies; tourists also visit some caves. The religious ceremonies occur seasonally and are associated with periodic pilgrimages. In Belize, the Rio Frio caves in the Cayo District are a popular tourist attraction and workers are known to sleep in the caves to guard against vandalism. Although bat guano is harvested in many Latin American countries and the United States, our study did not include locations where this occurs.

The parasite that causes Chagas disease, *Trypanosoma cruzi*, is transmitted by blood feeding triatomine insects [2]. These vectors are generally wide-ranging feeders and what they feed on is an important aspect of epidemiological risk; for example, human blood meals may mean increased risk of transmission to humans [3].

The species *Triatoma dimidiata* is currently the major vector of Chagas disease in Central America [4]. Some vector populations are found entirely in sylvan ecotopes, including caves; whereas others are nearly entirely domesticated and some move between ecotopes [5]–[9]. It is generally thought that sylvan vectors are not important for human transmission; however, few data are available to support this assumption. It is important to know if they are feeding on humans to help prevent transmission and to target control.

Because Chagas disease is the most economically important parasitic disease in Latin America with 8–10 million persons infected [10], we decided to investigate the potential role of cave dwelling *T. dimidiata* in transmission of *T. cruzi* to humans. To investigate the possibility that cave-dwelling, sylvatic, *T. dimidiata* contribute to the human transmission cycle, we determined the blood meal sources of *T. dimidiata* collected from caves in three regions of Central America where *T. dimidiata* is the main vector.

We tested the hypothesis that *T. dimidiata* collected from caves would not contain human DNA, and thus are not likely to be involved in transmission of *T. cruzi* to humans. In addition, we discuss the relationship between human cultural interactions with the cave and the blood meal sources found at each location.

## Methods

### Sampling locations and collection

Adult males and females were collected from inside caves in the departments of Petén and Alta Verapaz in Guatemala and Cayo district in Belize (Table 1). All the vectors had characters typical of the cave dwelling morph of *T. dimidiata* and are easily distinguished from those found in nearby houses and peridomestic areas by their longer, larger heads and reduced eyes and ocelli, as well as darker color for the adults and nymphs that are lighter in color [5], [11]. Wearing protective gear, five or six professionals from Laboratorio de Entomología Aplicada y Parasitología (LENAP), Universidad de San Carlos de Guatemala (USAC) collected the vectors. These personnel are trained in the safe handling of infectious agents and were experienced in searching for vectors. Personnel searched the walls, ceiling (when possible), and floor of the cave for 25 min, four times at each geographic location, with a 5–10 min. break between searches. Repeated searches are important because it takes vectors some time to emerge from hiding spots, presumably attracted by CO exhaled by the searchers. Triatomine nymphs in these caves are pale colored compared to nearby non-cave vectors, and have been observed to emerge from hiding in cracks and crevices of the cave walls and ceiling within a half hour following humans entering the cave. Adults are darker in color and easy to see in the caves because of the contrast with the limestone walls. Vectors were identified as *T. dimidiata* sensu lato based on published taxonomic keys [11].

**Table 1:** Cultural practices and anthropic alterations around the caves where the Chagas disease vector, *Triatoma dimidiata*, was collected.

Country	Guatemala			Belize
District or department	Petén	Alta Verapaz		Cayo
Municipality	San Luís	Lanquín	Cahabón	
Cave Name	Santa Isable	various	Santa María	Río Frío
latitude	17°03′24″N	15°34′50″N	15°36′20″N	16°58′43″N
longitude	89°59′30″W	89°48′22″W	89°48′45″W	89°00′23″W
landscape	steep hillside on privately owned cattle farm, access through thick brush	national park, large cave with many entrances and smaller caves, controlled access	deforested, small cave with one entrance for humans, not widely known, local residents monitor access	open area, sandy riverside beach
Closest human habitation	1400 m (landowner's house)	500 m (hotel and camp site)	600 m (two houses)	1200 m (military campground)
Artificial illumination	none	candles for religious ceremonies	candles for religious ceremonies	no evidence
Vegetation	pasture	forest	agriculture	forest
Livestock	cattle	free ranging livestock	free ranging livestock	none
Tourism	none	tourists - watch bats emerge from caves at dusk	none, cave is a 2 hour walk from the nearest road at the top of a hill	tourists - swimming
Other activities	Hunters, hunting dogs and possibly farm workers and feral dogs sleep overnight and shelter from the rain	seasonal Mayan religious ceremonies performed at night	seasonal Mayan religious ceremonies performed at night	working guards sleep in caves to guard against vandalism
type of blood meal source				
human	yes	yes	yes	yes
companion animal	yes	yes	yes	no
food animal	no	yes	yes	yes
wild animal	yes	no	no	yes
human commensal animal	yes	yes	yes	yes

The human cultural practices in the caves differ among locations. Near the municipality of San Luis, Petén, insect vectors were collected from a small cave located on a privately owned cattle farm. This particular cave was selected because the owner of the farm requested a survey to find the origin of vectors found in their home. Access to the cave is restricted and only local hunters and farm workers know of the cave; however, feral dogs from the nearby village may enter the cave in the rainy season. In Petén, hunting is a traditional weekend entertainment and caves are frequently used as overnight shelters. In this region, caves are not used for religious purposes and although hunters and their dogs may sleep and seek shelter in the caves, domesticated animals other than dogs are not brought to caves. There are no houses near the cave, it is surrounded by pasture areas for cattle.

In Alta Verapaz, caves near Lanquín and Cahabón were examined. Grutas de Lanquín, a complex of caves known to be infested with *T. dimidiata* [12] was chosen because the main cave is a tourist attraction and is used for Mayan religious ceremonies. The Lanquín River runs through the center of the cave. Thousands of bats inhabiting the cave provide a popular daily tourist attraction as they leave en masse at twilight. Ceremonial stone altars within the cave are still used by local residents for Mayan religious ceremonies that are performed at night. Candles and offerings to the gods are found inside the cave. There are several cave entrances but municipality guards control access. Human houses are located nearby.

Santa Maria cave, in the municipality of Cahabón, maintains the tradition of seasonal pilgrimages organized by descendants of the Maya. Travelers leave offerings to the gods in the cave, including sacrificial animals, pottery, candles, liquor, plant resins, and tamales. There are houses near the one main entrance to the cave, but these houses do not have domestic animals in captivity, and access to the cave is not controlled.

The caves at Rio Frio, Belize are a popular tourist attraction with several entrances to a wide open sandy area with the river flowing through the cave being a popular place for bathing. The ceiling is too high to search in this location, thus the walls are the main source of the vectors collected. Guards often sleep in these caves to protect against vandalism but there are no houses near the cave.

### DNA extraction, PCR amplification, cloning and sequencing

DNA was extracted from *T. dimidiata* abdomens as described previously [13], except we used the E.Z.N.A. Genomic DNA Isolation kit (Omega Bio-Tek, Norcross, GA). The *T. cruzi* infection status was determined for the vectors from Lanquin, Alta Verapaz using the TCZ primers following previously described methods [14], prior to starting the cloning procedure. Unfortunately, all of the extracted DNA from Rio Frio and San Luis Peten was used in the cloning assay and therefore we were unable to assess the *T. cruzi* infection status for the vectors from these locations. However a previous study found 25% *T. cruzi* prevalence in Peten [15].

The assay to identify blood meal sources was developed and previously reported by us [16]. Because the extractions of DNA from the insect abdomens potentially contain a mixture of blood meal DNA from multiple vertebrates, as well as insect DNA and parasite DNA (if infected), the initial PCR used primers specific for the mitochondrial 12S ribosomal RNA gene of vertebrates [17]. After confirming the PCR products were the expected size (∼215 bp), they were cloned using the pGEM-T Easy Vector System (Promega, Madison, WI, USA). Using the same 12S primers, cloning was verified by PCR, followed by sequencing in one direction with BigDye v3.1 (Applied Biosystems, Foster City, CA, USA) and analysis using an ABI PRISM 3730xl (Beckman Coulter, Fullerton, CA, USA). Sequence files were trimmed to 140 bp, edited using Sequencher v4.10 (Gene Codes Corporation, Ann Arbor, MI, USA) and taxonomic identification of the sequences was determined with a BLAST search using ≥99% identity as the criterion for a match.

### Statistical methods

To determine if feeding on the different blood meal sources varied among the three locations or between sexes, we used one way analysis of variance (ANOVA) for each taxa (humans, dogs, food animals, animals commensal with humans and wild animals). All statistical analyses were done using the JMP statistical package (JMP Version 10 [SAS Institute Inc., Cary, NC]).

## Results

**Figure 1:**
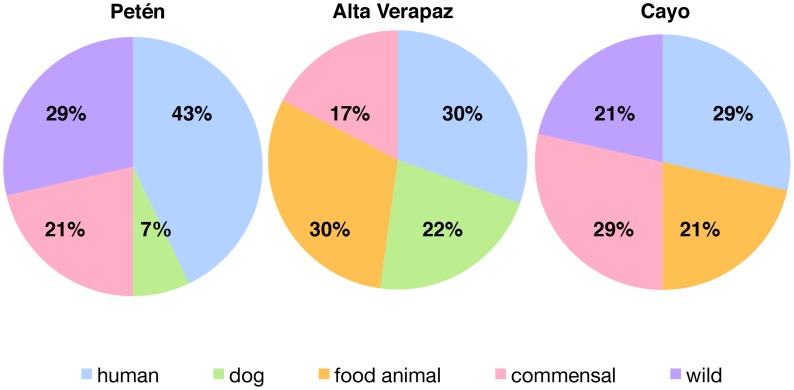
Ecological source of blood meals of *Triatoma dimidiata* collected from caves varies among locations. Vectors were scored as presence/absence for each ecological source. Data are percent of vertebrate host DNA by ecological source cloned from the abdomen of vectors from each location.

Human was the most common blood meal source in all three locations (Table 2, Figure 1), with over half the vectors from each cave feeding on human (Table 2: Petén = 8 of 9 vectors, Alta Verapaz = 7 of 10, and Cayo = 4 of 7). In Alta Verapaz, pig was equally abundant to human (both at 70%). Data on *T. cruzi* infection show that in Alta Verapaz, two of the three vectors that had human blood meals sources also had the parasite (Table 3).

**Table 2:** Blood meal sources of the Chagas disease vector, *Triatoma dimidiata*, collected from caves in three locations.

				Number of sequences of each taxa										
Cave - vector ID	sex	Season (R = rainy, D = dry), year	Taxa represented in blood meals	Human *Homo sapiens*	Dog *Canis lupus*	Pig *Sus scrofa*	goat or sheep Caprinae	Rat *Rattus norvegicus*	Mouse *Mus musculus*	Mallard duck *Anas sp.*	Vampire bat *Desmodus rotundus*	Hairy-legged bat *Myotis keaysi*	Opossum *Didelphis virginiana Philander sp*	opossum *Philander* sp.
Guatemala, Petén, San Luís, Santa Isabel (SI) cave														
SI - 1	F	R, 2008	3	1					1		10			
SI - 2	F	R, 2009	2						7				3	
SI - 3	M	D, 2007	2	1					11					
SI - 4	M	D, 2007	3	4	2								3	
SI - 5	M	R, 2009	1	10										
SI - 6	F	D, 2007	2	1									6	
SI - 7	F	R, 2008	1	7										
Average or %		9.6	2.0	86%	14%				43%		14%		43%	
Guatemala, Alta Verapaz, Lanquin, various caves														
Eunice-1	F	R, 2001	3	1		4	2							
Eunice-2	M	R, 2001	4	2	1	4	1							
Lobo-1	F	R, 2000	5		5	3	1	1	1					
Pony-1	M	R, 2001	3	2	1	1								
Tourist-1	M	R, 2001	2		5			2						
Guatemala, Alta Verapaz, Cahabón, Santa María (SM) cave														
SM – 1	F	R, 2001	3	1		4	1							
SM – 2	F	R, 2001	1					8						
SM – 3	F	R, 2006	4	2	2	4	1							
SM – 4	M	R, 2001	2	3				6						
SM – 5	M	R, 2006	3	2		4	1							
Average or %		7.6	3.0	70%	50%	70%	60%	40%	10%					
Belice, Cayo, Río Frío (RF) cave														
RF - 1	F	D, 2009	3	1		5	1							
RF - 2	F	D, 2009	3						1				6	1
RF - 3	F	D, 2009	1						11					
RF - 4	M	D, 2009	4	2		1				1			6	
RF - 5	M	D, 2009	4	2		1						4	4	
RF - 6	M	D, 2009	1					11						
RF - 7	M	D, 2009	3	3				1	7					
Average or %		9.9	2.7	57%		43%	14%	28%	43%	14%		14%	43%	14%

Blood meal sources were determined by PCR, cloning and sequencing of vertebrate 12S rDNA extracted from the vector abdomen.

**Table 3:** *Trypanosoma cruzi* infection status and blood meal sources of the Chagas disease vector, *Triatoma dimidiata*, collected from caves in Alta Verapaz, Guatemala.

Cave – vector ID	sex	*Trypanosoma cruzi* infection status	Human *Homo sapiens*	Dog *Canis lupus*	food animals	animals commensal with humans	wild animals
Eunice-1	F	−	+	−	+	−	−
Eunice-2	M	+	+	+	+	−	−
Lobo-1	F	−	−	+	+	+	−
Pony-1	M	−	+	+	+	−	−
Tourist-1	M	+	−	+	−	+	−
Santa María – 1	F	+	+	−	+	−	−
Santa María – 2	F	−	−	−	−	+	−
Santa María – 3	F	−	+	+	+	−	−
Santa María – 4	M	−	+	−	−	+	−
Santa María – 5	M	−	+	−	+	−	−
total		30%	70%	50%	70%	40%	0%

Overall we detected blood meals from eleven different taxa in the 24 vectors from the three locations, including duck and two species each of opossum and bat (Table 2). We examined a total of 218 sequences with 162 (74%) providing information on a blood meal source. On average, 9.04 clones were sequenced per vector, 7.11 sequences per vector were interpretable as a blood meal source, and individual vectors contained 2–3 blood meal sources (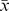 = 2.63, range 1–5). Using this small region of the 12S gene and the criteria of ≥99% match to a published vertebrate sequence, were able to determine the species for nine of the eleven taxa (human, dog, pig, mouse, rat, duck and the two bat species). We could not distinguish between goat and/or sheep, these two Bovids in the Subfamily Caprinae have identical 12S sequences for the regions examined. Similarly, we were only able to determine the subfamily for the two different opossum sequences (Didelphinae: *Didelphis sp.* or *Philander opossum*)

The non-human hosts fall into four groups that relate to human cultural interactions with caves: companion animal (dog), food animals (goat/sheep and pig), human commensal species (mouse and rat) and wild species (mallard duck and the two species each of bat and opossum). The mouse and rat species, *Mus musculus* and *Rattus norvegicus*, are human commensals and likely occur at these locations because of human activities. Humans and commensal animals were blood meal sources at all three locations (Figure 1). Food animals were not a blood meal source in Petén, likewise wild animals were not a blood meal source in Alta Verapaz and there was no evidence of feeding on dogs in Cayo.

Feeding on dog (F = 3.57; d. f. 2, 23; P<0.05), food animals (F = 5.56; d. f. 2, 23; P<0.02) and sylvatic animals (F = 4.68; d. f. 2, 23; P<0.03) were significantly different among locations. Feeding on humans (F = 0.64; d. f. 2, 23; P>0.05) and commensal animals (F = 0.23; d. f. 2, 23; P>0.05) did not vary among locations. There were no significant differences between males and females in the detection of human (F = 1.14; d. f. = 1, 22; P>0.05), dog (F = 0.05; d. f. = 1, 22; P>0.05), food animal (F = 0.22; d. f. = 1, 22; P>0.05), commensal animal (F = 0.001; d. f. = 1, 22; P>0.05), or sylvatic animal (F = 0.03; d. f. = 1, 22; P>0.05) blood meal sources.

The number of vectors feeding on bats was small, one each from Petén and Cayo and the bats were different genera. In contrast opossum are a more common blood meal source (43%, 3 of 7 vectors examined from both Cayo and Petén, including one vector from Cayo that had feed on both types of opossums). None of the vectors from Alta Verapaz had fed on opossum.

## Discussion

Our results indicate that cave dwelling, sylvatic *T. dimidiata* can contribute to the human transmission cycle. The evidence is that human was the most commonly found blood meal in *T. dimidiata* collected from caves. At the one location where we have *T. cruzi* infection data, we found 3 of 10 (30%) of vectors infected, and 2 of these 3 (2 of the 10 or 20% of vectors from that location) also had evidence of human blood. Human blood was found in >50% of all vectors in seven caves from three different locations. However, humans were not more common than the other blood meal sources combined. Of the total blood meals identified 27% at San Luis were from human (6 of 14 blood meal sources (43%), at Alta Verapaz 7 of 30 (23%), and at Rio Frio 4 of 19 (21%).

Further evidence of association with humans included substantial numbers of blood sources from the companion animal dog, domesticated food animals, and animals commensal with humans. Evidence of frequent human-bug contact was observed in the three different types of caves each with different ecological conditions and uses by humans. These results suggest a risk of transmission of *T. cruzi* to humans entering caves, however further study measuring the rates of *T. cruzi* infection in insects from all locations would clarify the transmission risk.

The high proportion of bugs that had evidence of human blood meals is surprising, given that caves are considered a sylvatic ecotope, and sylvatic vectors are generally thought not to be important in human transmission. Our results add weight to recent studies challenging this assumption. For example, in sylvan environments in Arizona and California, 38% of *Triatoma rubida* and *Triatoma protracta* had human blood sources [16], in *Triatoma infestans* in Bolivia 27% of blood meals detected were human [18]. However, a study of *Triatoma gerstaeckeri*, *Triatoma indictiva*, *Triatoma sanguisuga* and *T. protracta* in Texas found little evidence of feeding on humans [19]. Ecological factors including human encroachment into natural areas have been associated with emerging diseases worldwide [20].

The bugs from Santa Isabel Cave in Péten, a privately owned cattle farm with remnant forest patches, showed the highest incidence of human blood consumption (43%, Figure 1). We have anecdotal evidence from the owner of the farm that hunters and farm workers use the relatively cool cave for overnight sleeping or daytime resting, especially during the heat of the dry season. In addition, at the entrance of the cave we have found a clean wild animal skull, a common artifact at contemporary Maya hunting shrines [21], and machetes and plastic water bottles have been found inside the cave (M. C. Monroy, personal observation). Wild and human commensal animals were the next most preferred blood sources (29% and 21%, respectively). It is surprising that dog blood meals are rare (only 7%), since dogs usually accompany the hunters, but perhaps the dogs are eating bugs that come close [22]. Vectors from caves on this cattle farm had fed on the common vampire bat but interestingly had not fed on cattle, suggesting they do not leave the caves.

Humans and food animals are the main blood meal sources for *T. dimidiata* (both 30%) in the caves of Lanquín and Cahabón in Alta Verapaz, located within heavily deforested areas and surrounded by agricultural fields and houses (Figure 1). Frequent entry of human into the caves for tourism and religious ceremonies could explain the high proportion of blood meals from humans as these caves are popular destinations for tourists and Mayan religious ceremonies. The people performing the rituals may stay overnight in the caves; however, tourists are not likely to stay overnight in the caves, but rather camp near the caves (M. C. Monroy, personal communication or personal observation). Chickens, pigs, goats, and other food animals are free ranging in this area, which could explain high food animal blood sources (30%) in the bugs. The human companion animal dog (22%) and human commensal animals (17%) are the next most frequently found sources, and no wild animal blood sources were found in the bugs from these caves, demonstrating the strong association of humans and bugs in this area. Deforestation removes sylvan animal habitat and therefore sylvan blood sources. Anthropogenic change is known to alter blood meal sources [23].

Bugs found in Rio Frio, Cayo, Belize cave had fed mostly on human and human commensal animals (29% each, Figure 1). The cave is relatively small, has wide openings at both ends and is surrounded by forest. The high association with humans can be explained as the cave is a popular tourist attraction, frequented by swimmers, with guards often sleeping in the cave at night to protect against vandalism. Food animals and wild animals are the next most frequent blood source (21% each) which suggests it is a transit and resting place for wild and feral animals. For example, wild pigs have been reported in this area (http://www.belizeanway.com/destination-cayo). The bat fed upon in Cayo was most likely hairy-legged myotis (100% match to *Myotis keaysi*) but possibly elegant myotis (99% match to *Myotis elegans*) [24]. There was no evidence of dog blood meals in vectors collected in Rio Frio. The only nearby settlement is a British military base, and since the Belize military supervises the entrance to the cave it may be more restricted to hunters and their dogs.

Of the eleven taxa detected in the blood meals, about one-third were wild (five species total, duck, two opossum and two bat) and about one-third (7 of 24) of the vectors had fed on sylvatic animals. Due to land use practices, none of the vectors from Alta Verapaz had evidence of feeding on wild animals. The other locations showed similar wild animal blood sources: opossum was the most common (43% in both). In contrast, the number of vectors feeding on bats was small, one each from each locality (14%).

Although the vectors included in this study were all collected within caves, it is not known which of the blood meals we identified were actually obtained in the caves as it is possible the vectors could leave the caves to feed. However, our results are consistent with these vectors spending most or all of their lives in the caves. In Petén, although there is abundant cattle surrounding the caves, cattle blood was not found in the bugs. In addition several reports establish that *T. dimidiata* in caves in Central America differs morphologically from those found outside the caves suggesting they are different populations that do not intermingle [5], [25]. The bugs included in this study are the cave morphology. Therefore our results suggest the human-bug contact occurs when humans enter the cave. This may not be true everywhere as some *T. dimidiata* collected in houses in Colombia may be migrants from caves [5], [26] and in some localities *T. dimidiata* blood sources show evidence of migration between ecotopes [27]. Although a large number of different taxa (11) were detected in the abdomens of the 24 *T. dimidiata* this is likely a minimal estimate of blood sources as older blood meals may have been too degraded for detection [28].

Caves at all three locations had evidence of Chagas disease vectors feeding on humans and human commensal animals; as well as some combination of dogs, domesticated food animals and wild animals. The variation of blood meal sources among caves is understandable in the context of human uses of the caves at the three locations. We caution that human encroachment into natural areas may expose individuals to unanticipated risks of disease transmission.
